# From Strain Characterization to Field Authorization: Highlights on *Bacillus velezensis* Strain B25 Beneficial Properties for Plants and Its Activities on Phytopathogenic Fungi

**DOI:** 10.3390/microorganisms9091924

**Published:** 2021-09-10

**Authors:** Pierre Joly, Alexandra Calteau, Aurélie Wauquier, Rémi Dumas, Mylène Beuvin, David Vallenet, Julien Crovadore, Bastien Cochard, François Lefort, Jean-Yves Berthon

**Affiliations:** 1 Greentech, Biopôle Clermont-Limagne, 63360 Saint Beauzire, France; aureliewauquier@greencell.tech (A.W.); production@greencell.tech (R.D.); jeanyvesberthon@greentech.fr (J.-Y.B.); 2 LABGeM, Génomique Métabolique, Genoscope, Institut François Jacob, CEA, CNRS, Université d’Évry, Université Paris-Saclay, 2 Rue Gaston Crémieux, 91057 Evry, France; acalteau@genoscope.cns.fr (A.C.); mbeuvin@genoscope.cns.fr (M.B.); vallenet@genoscope.cns.fr (D.V.); 3 Plants and Pathogens Group, Research Institute Land Nature and Environment, Geneva School of Engineering, Architecture and Landscape (HEPIA), HES-SO University of Applied Sciences and Arts Western Switzerland, 1254 Jussy, Switzerland; julien.crovadore@hesge.ch (J.C.); bastien.cochard@hesge.ch (B.C.); francois.lefort@hesge.ch (F.L.)

**Keywords:** *Bacillus velezensis*, plant growth-promoting rhizobacterium, biocontrol agent, microbial ecotoxicology

## Abstract

Agriculture is in need of alternative products to conventional phytopharmaceutical treatments from chemical industry. One solution is the use of natural microorganisms with beneficial properties to ensure crop yields and plant health. In the present study, we focused our analyses on a bacterium referred as strain B25 and belonging to the species *Bacillus velezensis* (synonym *B. amyloliquefaciens subsp. plantarum* or *B. methylotrophicus*), a promising plant growth promoting rhizobacterium (PGPR) and an inhibitor of pathogenic fungi inducing crops diseases. B25 strain activities were investigated. Its genes are well preserved, with their majority being common with other *Bacillus spp*. strains and responsible for the biosynthesis of secondary metabolites known to be involved in biocontrol and plant growth-promoting activities. No antibiotic resistance genes were found in the B25 strain plasmid. In vitro and in planta tests were conducted to confirm these PGPR and biocontrol properties, showing its efficiency against 13 different pathogenic fungi through antibiosis mechanism. B25 strain also showed good capacities to quickly colonize its environment, to solubilize phosphorus and to produce siderophores and little amounts of auxin-type phytohormones (around 13,051 µg/mL after 32 h). All these findings combined to the fact that B25 demonstrated good properties for industrialization of the production and an environmental-friendly profile, led to its commercialization under market authorization since 2018 in several biostimulant preparations and opened its potential use as a biocontrol agent.

## 1. Introduction

Currently, agriculture is in a crucial transition phase leading to the gradual integration of new practices that take into account both socio-economic and environmental dimensions. In this context, the EU set rules for the sustainable use of pesticides in order to reduce their risks and impacts on human health and the environment (Directive 2009/128/EC). Therefore, agriculture is currently dealing with alternative trends due to the decrease of new synthetic products homologation and the reappraisal of many first-generation chemicals, enforced by the Water and Soil Framework directives (https://eur-lex.europa.eu/legal-content/EN/TXT/?uri=CELEX:32000L0060; https://www.elaw.org/content/eu-directive-200435ec-21-april-environmental-liability-regard-prevention-and-remedying-envir) (Accessed on 30 August 2021), the Biocide directive 98/8/EC (https://www.ecolex.org/details/legislation/directive-988ec-of-the-european-parliament-and-of-the-council-concerning-the-placing-of-biocidal-products-on-the-market-lex-faoc019011/) (Accessed on 30 August 2021), and the regulation Registration, Evaluation, Authorisation and Restriction of Chemicals (REACH) of the European Union (https://echa.europa.eu/regulations/reach/) (Accessed on 30 August 2021). One of the investigated and already recognized paths is the use of microorganisms as a solution to protect plants, by availing their beneficial properties [1]. Among them, bacteria with plant growth-promoting (PGPR) and/or disease-inhibiting (biocontrol) activities have become an important technological, economical, and political challenge. Many studies in the last decades focused on bacteria and especially on members of the *Bacillus* genus as candidates for their use in agriculture (for details, see [2]). 

Among them, *Bacillus velezensis* [3,4] (synonyms *Bacillus amyloliquefaciens* subsp. *plantarum* or *Bacillus methylotrophicus*) showed a wide range of beneficial properties on plants, environment and against phytopathogens, with the production of antimicrobial metabolites and the stimulation of the plant defenses [4,5,6]. Recent works emphasize that this bacterium may cumulate biostimulant and biocontrol properties, through a number of cluster genes for the synthesis of nonribosomal and ribosomal secondary metabolites, volatile compounds, and cyclic lipopeptides with antimicrobial action, also acting as stimulators for the induced systemic response (ISR) [7,8]. For instance, it was shown to promote the growth of *Malus hupehensis* while inhibiting the pathogen *Fusarium verticillioides* by secreting glucosidases [9]. The evaluation of four stains of this species also showed an efficient antagonistic effect against several major pathogenic bacterial and fungal species, including *Ralstonia solanacearum, Agrobacterium tumefaciens, Clavibacter michiganensis, Xanthomonas campestris*, *Alternaria brassicicola*, and *Sclerotinia sclerotiorum* [10], these effects being supported by the synthesis of several polyketides. Another work showed a reduction of *Agrobacterium fabrum* and *A. tumefaciens* populations in the rhizosphere of maize and tomato plants as well as the inhibition of crown gall symptoms in tomato and almond plants [11]. Interestingly, a strain of *B. velezensis* isolated from the mango phyllosphere showed a strong biocontrol effect against the fungal agent of mango anthracnose, while acting as an efficient biostimulant on maize and *Arabidopsis* [12]. Some strains of *B. velezensis* may display very strong antifungal activity through peroxidases, cellulases, and proteases such as strain ZW-10 efficient against *Magnaporthe oryzae* the fungal agent of rice blast, a major disease of rice culture [13]. These few and diverse recent examples are a short sample of the ca. 2200 papers involving *Bacillus velezensis* since 2020. This species and its numerous strains therefore represent promising agents with biostimulant and biocontrol activities. Genomic analysis and genetic engineering of this species are already investigated to improve their action [5,14,15].

In the present study, we report the first results in terms of beneficial properties of the *Bacillus velezensis* strain B25, whose genome has been made available [16]. B25 is a Gram-positive bacterium isolated in Switzerland from the inner wood tissues of a decaying *Platanus* X *acerifolia* tree. It is a close neighbor of the well-studied PGPR strain FZB42 (more than 2000 articles since its first description by Krebs et al. [17]) in terms of gene content comparisons and synteny conservations. This work brings an overview of the B25 strain specificities in terms of genomic features while giving first conclusions on its abilities as a PGPR and potential biocontrol agent of several major phytopathogenic fungi of cereal crops. As the current European regulation 2009/1107 EC for the registration of new active substances requires data on their environmental impacts, we also investigated this point on two major representatives of soil and water ecosystems. Conclusions are drawn in order to address *Bacillus velezensis* strain B25 legitimacy as a future biocontrol agent.

## 2. Materials and Methods

### 2.1. Bacillus velezensis Strain B25 Genomic Features Assessment

#### 2.1.1. Genome Characterization

The genome of *B. velezensis* strain B25 [16] (accession no. LN999829 and LN999830) was integrated in the MicroScope platform [18,19] to perform its annotation and comparative analyses. Core pangenome genome computations were performed using the following MicroScope gene families (MICFAM) parameters 80% amino acid identity/80% alignment coverage.

A species tree was generated by a concatenation of nine housekeeping genes: gyrA, dnaX, glyA, cysS, glpF, gmk, gyrB, ligA, and recN. Homologs of each gene were identified using the Basic Local Alignment Search Tool of the National Center for Biotechnology Information (https://blast.ncbi.nlm.nih.gov/Blast.cgi) (Accessed on 30 August 2021), for BLAST searches in the genomes of 16 *Bacillus amyloliquefaciens* strains, 2 *B. subtilis* strains (168 and *subsp*. *spizizenii* W23), *B. atrophaeus* strain 1942, 2 *B. cereus* strains ATCC 10987 and ATCC 14579, and *B. licheniformis* strain ATCC 14580. They were aligned using the software Multiple Alignment using fast Fourier transform (MAFFT) v.7.307 (default parameters; [20]). Ambiguous and saturated aligned regions were removed using the Block Mapping and Gathering with Entropy (BMGE) software v.1.12 (parameter gap rate set to 0.5; [21]). The resulting nine housekeeping genes sequences per genome were then concatenated respectively. A maximum-likelihood phylogenetic tree was generated with the alignment using the phylogeny software based on the maximum-likelihood principle PhyML v.3.1.0.2 [22] and the generalized time reversible (GTR) substitution model with gamma-distributed rate variation (six categories), estimation of a proportion of invariable sites and exploring tree topologies using the best tree option (BEST). One thousand bootstrap replicates were performed.

#### 2.1.2. Gene Analysis

Antibiotic resistance gene resistance analysis was conducted with the Resistance Gene Identifier of the Comprehensive Antibiotic Resistance Database CARD/RGI (v.3.1.2/5.2.0) system [23] and the Antimicrobial Resistance Gene Finder Plus software (AMRFinderPlus (NCBI) v.3.10.5 and database version 2021-06-01.1) [24]. A comparison was carried out with 20 other *Bacillus methylotrophicus* genomes from the MicroScope platform for these genes. Nonribosomal peptide synthetases and polyketide synthases (NRPS/PKS) gene clusters were identified using the software Antibiotics and Secondary Metabolite Analysis Shell (antiSMASH6) [25]. Each prediction was analyzed manually to identify clusters coding for already described compounds and to check if the gene clusters were complete or not. The borders of each cluster were manually refined while checking for synteny among genomes using the MicroScope platform interface. 

A search for genes of polysaccharide-degrading enzymes, including the major cell wall-degrading enzymes (CWDEs, i.e., chitinase, xylanase, and β-glucanase) involved in mycoparasitic activities, was also run through the web server for automated carbohydrate-active enzyme annotation (dbCAN) [26].

### 2.2. Assessment of the Inhibition of Fungal Pathogens by Bacillus velezensis Strain B25

#### 2.2.1. B25 Culture Conditions

B25 was cultivated on tryptic soy agar (TSA) slants at 30 °C for 3 days. Bacteria were then suspended in 9 mL of isotonic diluent (casein pepton 2 g; NaCl 9 g; Tween80 5 mL; qs 1 L). The bacterial suspension was mixed with sterile glycerol (3.5:1) and stored at −80 °C until use.

#### 2.2.2. In Vitro Confrontations for Antagonism Potential Evaluation

Thirteen phytopathogenic fungal strains from Greencell (France) corporate collection were used for in vitro confrontations. These include *Fusarium culmorum* strain B376; *Fusarium graminearum* strains B375, B377, FG183 B381, FG171 B382, B383, and FG155 B385; *Fusarium moniliforme* strain B378; *Fusarium verticillioides* strains FV63 B384 and FV838 B386; *Gaeumannomyces graminis* strain B278; *Microdochium nivale* strain B379; and *Septoria nodorum* strain B380. Strains were cultivated on potato dextrose agar (PDA, Difco-France) Petri dishes at 25 °C until sporulation. 

In vitro challenge experiments were conducted by adapting the method described by Denis et al. [27]. Agar disks (diam. 5 mm) from fungal cultures were cut and removed from the growing borders of the colonies and transferred to the center of another PDA Petri plate (Ø 140 mm). Fungi were grown for 3 days at 25 °C to obtain a 20 mm diam. colony. Then, four non-impregnated sterile paper disks (Oxoid Antimicrobial Susceptibility Discs, diam. 5 mm) were placed on the medium at 45 mm from the center in order to form two perpendicular axes, which cross point is the pathogen colony. One axis was for Control (C1 and C2 discs; inoculated with 15 µL of sterile water), the other was for strain B25 Treatment (T1 and T2 discs; inoculated with 15 µL of bacterial suspension). The experiment was run for 15 days, with plates incubated at 25 °C, and conducted in three replications for each pathogenic fungus. 

To calculate inhibition indexes, assessments of pathogen growth (mm) were performed with four measurements: *radC*_1_ = pathogen radius in front of C1; *radC*_2_ = pathogen radius in front of C2; *radT*_1_ = pathogen radius in front of T1; *radT*_2_ = pathogen radius in front of T2. Inhibition indexes were calculated at different days depending on the pathogen growth, to follow their evolution throughout the experiment, with:

(1) $$\mathrm{Inhibition}\;\mathrm{index}\;(\mathrm{II})=\frac{\left(mean\;radC-mean\;radT\right)}{45}$$

Antagonism interaction is confirmed when the inhibition index reaches the threshold of 0.5. Additionally, daily observations were made to identify potential inhibition mechanisms.

#### 2.2.3. Bioassay in Growth Chamber for Biocontrol Evaluation against *F. graminearum* Strain B377

*Fusarium graminearum* (teleomorph *Gibberella zeae*) strain B377 (kindly provided by UR 1264 MycSA INRA Bordeaux, France) was used in growth chamber bioassays. The strain was propagated on PDA Petri plates and maintained at 4 °C. Spore suspensions were generated by inoculating 15 agar plugs in 150 mL of CMC medium (C4888, Sigma–Aldrich, St. Quentin Fallavier, France) and incubated at 25 °C and 150 rpm for 3 days. After filtration through Sefar Nitex 03100 (100 μm, SEFAR AG, Heiden, Switzerland), the spores were counted on Thoma cell counting chamber before suspension in water at the defined concentration of 6.5 × 10^5^ colony forming units (cfu)·mL^−1^.

Two similar independent bioassays were carried out. The experiments were conducted in randomized complete block design with 30 plants per treatment for 3 weeks. Experimental conditions consisted of treated plants of wheat cultivar “Soissons” with (i) B25, (ii) B25 and B377, (iii) B377, (iv) sterile distilled water.

Seeds were surface-sterilized prior to treatment. Briefly, seeds were shaken in 5% sodium hypochlorite solution for 3 min, rinsed three times in sterilized distilled water and dried on sterile filter paper. Then the seeds were individually sown in small culture pots (5 cm in diameter × 6 cm high) filled with 21 g of Substrat 5 Perlite culture medium (Klasmann–Deilmann, Bourgoin Jallieu, France). 

The *Bacillus velezensis* B25 inoculation was performed at day 0 with 200 μL of the strain at 2 × 10^7^ cfu·mL^−1^ in each pot. Control treatments were inoculated with 200 μL of sterile distilled water.

The *Fusarium graminearum* B377 inoculation was performed at day 4 with 1 mL of cell suspension at 6.5 × 10^5^ cfu·mL^−1^ in each pot. Control treatments were inoculated with 1 mL of sterile distilled water.

Plants were incubated in growth chamber with fixed parameters (25 °C; 75% relative humidity; 16/8 day/night cycle with a light intensity of 360 μmol m^−2^·s^−1^). To avoid desiccation, pots were watered every two days with 20 mL of sterilized distilled water. 

Damping-off symptoms on crown and root rot were recorded macroscopically at the end of the experiments (day 21). Effects of the different treatments were assessed by dry weight measurements of aerial and root parts of the plants.

### 2.3. Bacillus velezensis Strain B25 Metabolic Properties Assessment

#### 2.3.1. Phosphate Solubilization Assay

The assay was performed as previously described [28]. Briefly, the National Botanical Research Institute’s phosphate-bromophenol blue (NBRIP-BPB) liquid medium was prepared and adjusted to pH 7. Flasks containing 10 mL of NBRIP-BPB medium were inoculated with a 1% (*v*/*v*) inoculum from B25 pre-cultures grown in tryptic soy broth (TSB). The flasks were incubated for 3 days at 30 °C on a shaker set at 180 rpm. A flask containing non-inoculated medium served as control.

After incubation, supernatants were recovered by 10 min centrifugation at 10.000 rpm. The final supernatants OD_600nm_ were measured and a percentage of OD_600nm_ diminution was calculated as follows:

(2) $$\%{\mathrm{OD}}_{600\mathrm{nm}}\;\mathrm{diminution}=1-\frac{{\mathrm{OD}}_{600\mathrm{nm}}\;\mathrm{culture}\;\mathrm{supernatant}}{{\mathrm{OD}}_{600\mathrm{nm}}\;\mathrm{control}}\times 100$$

A quantitative assay was conducted by ICP-radial on the supernatants.

#### 2.3.2. Siderophores Detection Assay

The assay was performed by using the overlaid Chromeazurol S (O-CAS) agar medium [29]. Glasswares were washed with HCl 6M to get rid of iron traces.

B25 strains were spotted on a rich iron-depleted solid plate count agar medium (PCA, Thermo Fisher Scientific, Geneva, Switzerland) then incubated 3 days at 28 °C. After incubation, cultures were overlaid with O-CAS medium (10 mL for 90 mm diam. Petri dishes) and incubated at 28 °C [30].

The plates were checked for a discoloration halo formation around colonies: siderophores production is characterized by color change of the medium (orange, yellow, or rose) according to the type of siderophores produced.

Control plates of non-inoculated O-CAS-agar were incubated under the same conditions. 

#### 2.3.3. Indolacetic Acid (IAA) Production Assay

The Salkowski method to determine concentrations of IAA in supernatants was used, as previously described [31] and according to Gordon and Weber [32].

Briefly, tryptic soy broth (TSB) was used for B25 pre-cultures. The strain was cultivated at 30 °C for 3 days on a shaker set at 180 rpm. A modified TSB, supplemented with 1 g·L^−1^ L-Tryptophan, was used as specific liquid medium to induce auxin-like phyto-hormones production by the bacteria. Flasks containing 10 mL of TSB-L-Trp each were inoculated with 10% (*v/v*) of bacteria inoculum from pre-culture and incubated 2 days at 30 °C on a shaker at 150 rpm. Cultures were centrifuged for 15 min at 5000 rpm to recover supernatants. 

For the calibration curve, standard solutions ranging from 0 to 20 µg·mL^−1^ were prepared from an IAA stock solution (Sigma–Aldrich, St. Quentin Fallavier, France^®^) at 2 g·L^−1^. IAA production was assessed by mixing samples (supernatant or standard) to Salkowski reagent (*v*/2*v*). After 30 min incubation in the darkness, OD_535nm_ was measured, and IAA production was assessed by comparison with the calibration curve.

#### 2.3.4. Metabolic Profiling on Biolog GenIII Microplates

To determine the metabolic profile of B25, an analysis on 71 carbon sources and 23 chemical products was carried out, using Biolog GenIII™ microplates (Biolog Inc., Hayward, CA, USA). These microplates, developed for the identification of microbial isolates, are based on the substrates utilization profiles. The 96 micro-wells contain varied carbon or chemical substrates mixed with tetrazolium violet dye. The appearing of a blue purple coloration indicates that the substrate has been metabolized and that the related enzymatic activity was expressed. The inoculation of Biolog GenIII microplates was carried out following the prescriptions of the manufacturer. After 48 h incubation at 28 °C, the microplates were read, using an absorbance microplate reader ELx800™ (BioTek Instruments, Inc., Winooski, VT, USA) and the turbidity was measured at 590 nm.

#### 2.3.5. Antibiograms and Minimal Inhibitory Concentrations Evaluation

In order to determine the minimal inhibitory concentrations (MICs) of the seven antibiotic classes (β-lactamine, aminoglycoside, cephalosporin, quinolone, glycopeptide, macrolide, and tetracycline), antibiograms were performed using Oxoid MIC Evaluator™ strips (Thermo Fisher Scientific, Basingstoke, UK). For each antibiotic, of each class (Imipenem, Amikacin, Ceftazidime, Ciprofloxacin, Vancomycin, Erythromycin, Tetracycline, respectively), MICs were measured twice. A fresh starter culture was carried out in Luria Bertani (LB; Roth, Switzerland) broth and incubated at room temperature for 48 h. Then, bacterial mats were established on Petri dishes containing LB agar, by plating 200 µL of liquid culture per plate. MIC evaluator strips were then laid on the agar medium following the manufacturer’s recommendations. After 48 h incubation at room temperature, the MIC of each antibiotic was directly read on MIC evaluator strips.

#### 2.3.6. Growth Response under UV-C Stress

To estimate the effect of UV-C, over an exposition time range of 0 to 30 min, on the growth of strain B25 and a commercial strain of *Bacillus amyloliquefaciens*, IT45 (Lallemand Plant Science, Toulouse, France), a UV-C resistance test was performed under a laminar flow. The lighting source used was a Philips neon tube TUV TL-D 30W G13 (wavelength 250 nm). A starter culture, in 10 mL of medium LB broth, was incubated at room temperature for 48 h. Each bacterial strain was spread on seven Petri dishes (one Petri dish per exposure time), containing LB agar. After exposure to UV-C lighting, Petri dishes were incubated at room temperature for 48 h. The results were scored and expressed by presence (+) or absence (−) of growth. 

### 2.4. Bacillus velezensis Strain B25 Ecotoxicological Impact on Representative Organisms

#### 2.4.1. Effects on Mortality and Reproduction of *Eisenia fetida*

To estimate the potential effect of B25 after soil applications, a study on a soil representative organism, the earthworm *Eisenia fetida*, was performed, according to ISO 11268-2 standard. Briefly, the B25 strain, formulated in dextrose powder at a concentration of 10^8^ cfu g^−1^, has been incorporated into artificial soil at the following doses: 1.33; 2.66; and 13.3 mg of test element kg^−1^ of dry soil, corresponding to 1, 2, and 10 times the maximum annual use rate of the currently sold product Heles^®^ (Greencell) containing the B25 strain (assuming an average soil depth of 5 cm and a density of 1.5 kg·L^−1^).

The direct effects of treatment (mortality and effects on growth) were evaluated after 28 days. Reproduction was assessed at the end of the second four-week period after treatment, by counting the number of cocoons and juveniles present in all replicates.

#### 2.4.2. Chronic Toxicity Effect on *Daphnia magna*

The method used followed the Guidelines for Testing of Chemicals (2012) No 211 of the Organisation for Economic Co-operation and Development, (OECD) “*Daphnia magna* Reproduction Test” also referenced as Method C.20 of the European Commission Regulation (EC) No. 440/2008. Briefly, *Daphnia magna* individuals were exposed (10 replicates of a single daphnid per group) to solutions of B25 strains equivalent to 4.5 × 10^7^, 1.0 × 10^8^, 2.2 × 10^8^, 4.5 × 10^8^, and 1.0 × 10^9^ cfu·L^−1^ respectively, for a period of 21 days.

The numbers of live and dead adult waterfleas and young daphnids (live and dead) were determined daily. The daphnias were fed daily with a mixture of algal suspension and Tetramin^®^ flake food suspension.

### 2.5. Statistical Analysis

For the biocontrol bioassay, the experiment was repeated twice, and statistical analysis of variance (ANOVA) and Tukey comparisons were made using R software (v3.2.1). 

## 3. Results

### 3.1. Characterization of the General Genomic Features of Strain B25

#### 3.1.1. Genome Specificities

The main genomic features of *B. velezensis* strain B25 are summarized in Table 1 and compared to 4 closely related strains. Its circular chromosome of 3.854.619 bp is the shortest of these *Bacillus* strains. Its G+C content is higher than *B. subtilis* 168 but close to *B. methylotrophicus* FZB42, *B. amyloliquefaciens* IT-45, and *B. licheniformis* ATCC14580. The number of predicted coding regions (3679) is lower than in the 4 other strains, with the strain ATCC14580 having the highest number of CDS. Conversely, the average size of CDS (934) is bigger for strain B25 than for the others. B25 genome also presents 68 tRNAs and 7 copies of 5S, 16S and 23S rRNA.

**Figure 1:**
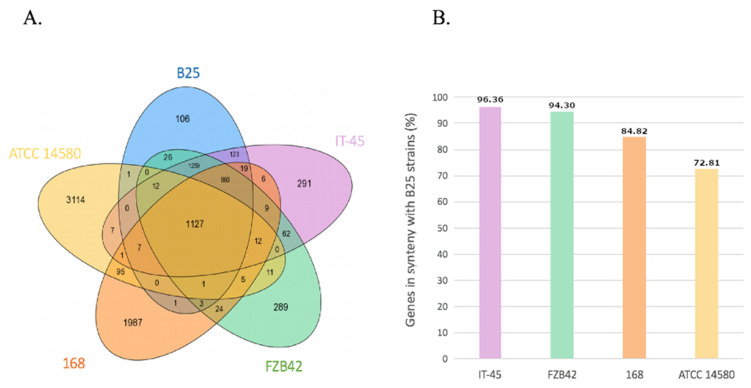
Comparisons of *Bacillus velezensis* strain B25 genome with four other *Bacillus spp.* (*B. methylotrophicus* FZB42, *B. amyloliquefaciens* IT-45, *B. subtilis 168* et *B. licheniformis* ATCC 14580). (**A**) Venn diagram of genome-specific or common genes between B25 and the other strains. (**B**) Percentage of genes in conserved synteny between B25 and the other strains.

The majority of B25 genes are shared with at least one other strain, with only 106 specific coding genes (2.9%, mostly pseudo-genes and unknown genes; Figure 1A). As depicted in Figure 1B, *B. amyloliquefaciens* IT45 is the most similar to B25 in terms of synteny conservation (96.36%). However, the majority of B25 genes are in conserved synteny with *B. subtilis* (3234 genes, 86.6%) and *B. licheniformis* (2896 genes 77.5%), respectively.

#### 3.1.2. Phylogenetic Relationship of Bacillus velezensis Strain B25

**Figure 2:**
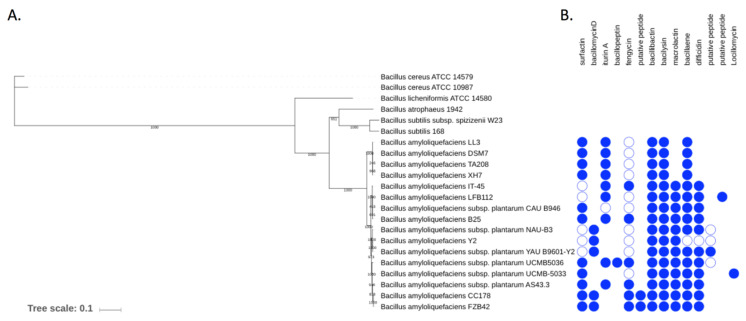
Distribution of NRPS/PKS gene clusters in *Bacillus amyloliquefaciens* strains in relation to their phylogeny. (**A**) Position of *B. velezensis* B25 in relation to other species within the genus *Bacillus*. The species tree was generated by a concatenation of nine conserved proteins using a Maximum Likelihood method for 22 genomes. Bootstrap values above 80% are indicated by black dots. (**B**) Pattern of presence/absence of NRPS/PKS gene clusters for 16 *B. amyloliquefaciens* strains. The presence of a gene cluster for a given compound is indicated by a circle shape, which is filled in blue when the cluster is complete or empty when the cluster is partial.

The phylogenetic tree clustered mainly 3 groups of *Bacillus* (Figure 2), with a major group (Cluster 1) including all *B. amyloliquefaciens* strains (16 entries, including strain B25) being the closest to another group composed of *B. atrophaeus* and *B. subtilis* strains (Cluster 2). *B. licheniformis* ATCC 14580 and then *B. cereus* group (Cluster 3) were the strains coming afterward in the tree.

#### 3.1.3. Genes Involved in Biocontrol

*B. velezensis* B25 genome harbors gene clusters involved in the biosynthesis of secondary metabolites known to display biocontrol and plant growth promoting activities (Table 2). These gene clusters correspond to modularly organized mega-enzymes defining both nonribosomal peptide synthetases (NRPS) and polyketide synthase (PKS). Five NRPS coding for surfactin, mycosubtilin, fengycin, bacillibactin and bacilysin, two PKS coding for macrolactin and difficidin, one ribosomal peptide and one NRPS/PKS coding for bacillaene were found in B25 genome. These genes were previously described and presented a high similarity with FZB42 strain (for 7 genes, [33]), mycosubtilin was similar to strain IT-45 [34] and amylolisin to *B. methylotrophicus* strain GA1 [37].

#### 3.1.4. Detection of Resistance Genes to Antibiotics

Several potential antibiotic resistance genes were identified after the analysis of the B25 genome (Table 3). They encode different antibiotic resistance proteins, including those for tetracycline and penicillin. 

The resistance genes present in B25 are also found in the majority of the *Bacillus methylotrophicus* strains analyzed and in the strains IT45, FZB42, and 168. No resistance gene was found in the plasmid of the B25 strain. 

#### 3.1.5. Polysaccharide Degrading Enzymes 

The analysis of B25 genome unraveled the presence of different glycosidase enzymes from the hydrolase family, including xylanase and glucanase enzymes (Table S1).

### 3.2. Characterization of the Biocontrol Properties of Bacillus velezensis Strain B25

#### 3.2.1. In Vitro Microbial Interactions Tests

*Bacillus velezensis* strain B25 antagonistic effect was assessed in vitro against thirteen fungal pathogens (Figure S1). These tests showed the efficiency of the bacteria to fight against 9 of these fungi (B376, B377, B378, B379 B380, B382, B384, B385, and B386 strains). A rather distinct circular antibiosis area between both colonies was observed. Moreover, pathogenic mycelium edge was thinner and seemed to present an area with a more important sporulation and a deeper coloration. 

Some pathogenic strains (B375, B381 and B383), however, were very sensitive to *B. velezensis* strain B25, with weak antibiosis phenomenon. 

**Figure 3:**
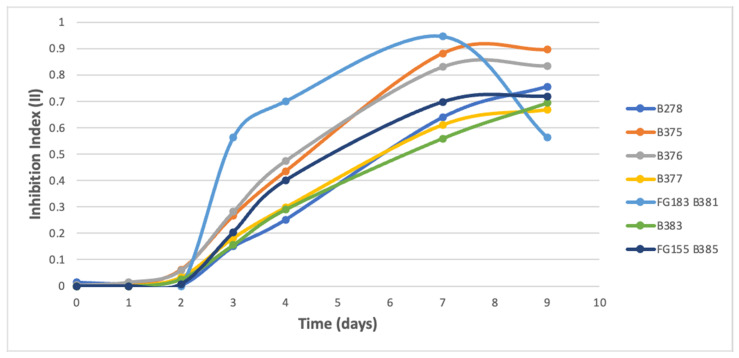
Kinetic curves of global inhibition indexes from the challenge co-cultures of *Bacillus velezensis* B25 against pathogenic fungi *Gaeumannomyces graminis* B278; *Fusarium graminearum* B375; *Fusarium culmorum* B376; *Fusarium graminearum* B377; *Fusarium graminearum* FG183 B381; *Fusarium graminearum* B383 and *Fusarium graminearum* FG 155 B385.

**Figure 4:**
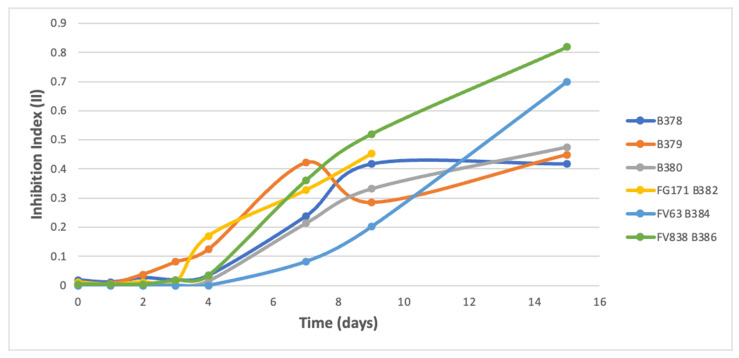
Kinetic curves of global inhibition indexes from the challenge co-cultures of *Bacillus velezensis* B25 against pathogenic fungi *Fusarium moniliforme* B378; *Microdochium nivale* B379; *Septoria nodorum* B380; *Fusarium graminearum* FG171 B382; *Fusarium verticillioides* FV63 B384; and *Fusarium verticillioides* FV838 B386.

Inhibition indexes (II) results were followed during 9 to 15 days. A latency phase was noticed during the first days of microbial growth, followed by two types of evolution curves for the indexes:

(i) A fast increase of the II was observed for the B25 challenge co-cultures against B278, B375, B376, B377, B381, B383, and B385 (Figure 3); with a 2-day latency phase followed by an exponential-like increase in 7 days, and finally a plateau with II reaching 0.7532, 0.8970, 0.8107, 0.6850, 0.6010, 0.6898, and 0.7243, respectively. For B381 strain, the plateau was not the maximum II reached (0.9626 at 7 days of culture followed by a decrease to the stationary phase). It could be explained by a strong antibiosis of B25 until 7 days before another phase of pathogen growth, however insufficient to resist B25.
(ii) A longer latency phase (4 days) followed by a gradual increase of the II until the end of the experiment (15 days) was observed for the B25 challenge co-cultures against B378, B379, B380, B382, B384, and B386, with maximum II reaching 0.4052, 0.4516, 0.4663, 0.6010, 0.6854, and 0.8157, respectively (Figure 4).

#### 3.2.2. In Planta Biocontrol Activities

In planta experiment on wheat cultivar “Soissons” was carried out to evaluate the biocontrol effect of *B. velezensis* strain B25 against fusarium seedling blight (FSB). As shown in Table 4, *F. graminearum + B. velezensis* treatment tend to reduce the FSB disease severity in test 1 of approximately 30% and significantly succeed to reduce it of almost 50% in test 2, in comparison with the *F. graminearum* treatment alone. No particular effect on the root and stem length was observed, whatever the treatment (data not shown).

### 3.3. Characterization of the Metabolic Properties of Strain B25

#### 3.3.1. Phosphate Solubilization

**Figure 5:**
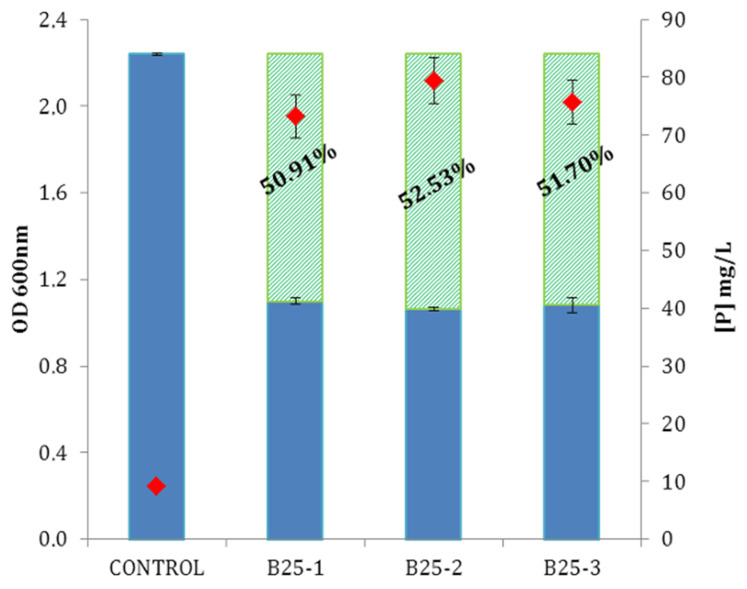
Phosphorus solubilization ability of *Bacillus velezensis* B25 compared to control in 3 independent experiments (B25-1, B25-2, and B25-3): OD_600nm_ (blue columns); percentages of OD_600nm_ decrease of B25 cultures compared to control (green columns); phosphorus rates in culture supernatants in mg·L^−1^.

Phosphate solubilization is characterized by an OD_600nm_ of the culture supernatant inferior to the control condition_._ As BPB is a pH indicator, a comparison of OD_600nm_ from cultures and control laid the stress on the strain ability to produce organic acids in its environment. A color change was noticed as the OD_600nm_ in the inoculated NBRIP-BPB flask decreased (average of 51.71%, Figure 5). 

ICP-Radial measurements of soluble phosphate rates available in supernatants of culture assays showed an average of 76.10 mg/L of available [P] compared to the 9.17 mg/L available in control condition which represent an increase of 830% (Figure 5). 

#### 3.3.2. Siderophores Detection

O-CAS solid medium revealed that B25 strain produced hydroxamate-type siderophores after 24 h of incubation, represented by orange halos appearing around the colonies (Figure S2). 

#### 3.3.3. IAA Production

**Figure 6:**
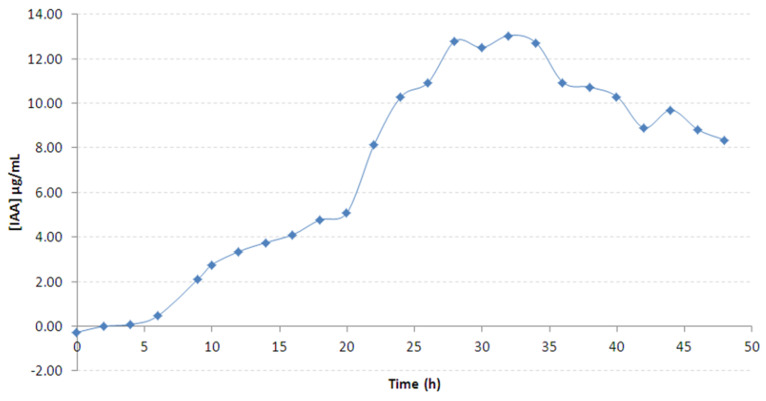
Production of auxins-type phytohormones (equivalent IAA) by *Bacillus velezensis* B25, during 48 h in TSB+L-Trp broth.

IAA production of B25 was monitored for 2 days (Figure 6). The production of IAA increased slowly to reach 5.099 µg·mL^−1^ between 4 and 20 h. Afterwards, the production increased faster and reached a maximum of 13.051 µg·mL^−1^ at 32 h). IAA decreased then slowly and reached a quantity of 8.347 µg·mL^−1^ at the end of the culture.

#### 3.3.4. Metabolic Profiling on Biolog GenIII Microplates

Table 5 summarizes the metabolic profile obtained for B25 with the use of Biolog GenIII microplates. It appears that B25 is able to metabolize and grow on most single carbon sources such as glucides and amino acids, although not able to grow on *P*-hydroxyphenylacetic acid, α-hydroxybutyric acid and α-ketobutyric acid. It does not metabolize or is sensitive to the following antibiotics: fusidic acid, troleandomycin, rifamycin SV, minocycline, nalidixic acid, and vancomycin. It is also sensitive to the detergent Niaproof 4, but resistant to the antibiotic lincomycin and to the chaotropic agent guanidine HCL. Furthermore, it grows up to 8% NaCl and at pH5 and pH6. Finally, it is able to grow on tetrazolium violet and sodium butyrate but not on tetrazolium blue and sodium bromate. This metabolic profiling provides with important information related to possible usable carbon sources and growth conditions for production as well as for chemical agents able to control B25 growth.

#### 3.3.5. Minimal Inhibitory Concentrations (MICs) of Antibiotics

MICs are given in Table 6. They vary between 0.015 (Imipenem) and 32 µg·mL^−1^ (Ceftazidime) and show that B25 is sensitive to all antibiotics used here at the exception of ceftazidime, a cephalosporin, to which it is slightly resistant.

#### 3.3.6. UV Sensitivity

*Bacillus velezensis* strain B25 proved to be less UV sensitive than *B. amyloliquefaciens* IT45 (Lallemand Plant Science) since it still survives after 10 min exposure to UV-C, while IT45 does not survive at all after 5 min exposure. The results are summarized in Table 7. 

### 3.4. Characterization of the Ecotoxicological Impact of Strain B25

The studies performed on *Eisenia fetida* and *Daphnia magna* revealed the absence of effects of strain B25 on these organisms, even at doses far exceeding the rate of application of the commercial product Heles^®^ (1 × 10^8^ cfu·g^−1^ of product; rate of single application 500 g·ha^−1^, maximum 2 applications per year). 

For *Eisenia fetida*, the dosage for both no observed effect concentration (NOEC) and half maximal effective concentration (EC_50_) was set at 10 kg·ha^−1^, corresponding to 10-times the highest annual rate of use for this product (Table 8).

Exposure of *Daphnia magna* to the test item gave results based on the nominal test concentration (Table 9). Results set the NOEC at 2200 mg·L^−1^ (equivalent to 2.2 × 10^8^ cfu·L^−1^) for body length parameter, and at 4500 mg·L^−1^ (equivalent to 4.5 × 10^8^ cfu·L^−1^) for immobilization and reproduction parameters.

## 4. Discussion

*Bacillus velezensis* strain B25 is already present on the European market (EU and Switzerland) in agronomical products for plant stimulation. As many other *Bacillus* strains, it possesses features commonly highlighted for plant stimulation, such as phosphate solubilization capacities and production of siderophore and auxins [5,38]. Phylogenetically, strain B25 is a close neighbor of *Bacillus amyloliquefaciens* IT-45 [34], another strain authorized on market for the same purpose. Independently of their potential to colonize rhizosphere (which has not been evaluated in this study), strain B25 expressed a higher resistance to UV-C exposure, which could be seen as an advantage for application on aerial plant parts, giving it a better chance to survive in this particular case of use. This might open the way to foliar treatments against foliar pathogens such as the ones tested in this study or against the ubiquitous pathogen *Botrytis cinerea* against which some strains of *B. velezensis* have already shown a certain biocontrol efficiency [39,40]. In fact, this higher resistance might be due to the physiological state of the strain, as it has already been highlighted that the dormant spore forms of *Bacillus* are 5 to 50 times more resistant to UV radiation compared to the corresponding growing cells [41]. Production of *Bacillus sp.* strains under its spore form therefore seems to be an essential step for the marketing of a *Bacillus*-based product.

*Bacillus velezensis* strain B25 set of genes clusters are involved in the synthesis of a wide range of secondary metabolites known to have plant stimulation and pest control activities. Interestingly, these metabolites are shared by other *Bacillus* strains [33,34,35,36,37], but this particular association (i.e., surfactin, fengycin, bacillibactin, bacilysin, macrolactin, bacillaene, difficidin, mycosubtilin, amylolysin) has not been described in currently sequenced *Bacillus* strains. 

Laboratory experiments with in vitro antagonistic microbial interaction tests showed the capacity of the strain B25 to control a broad range of plant pathogens, essentially cereal pathogens, belonging to the genera *Gaeumannomyces*, *Fusarium*, *Microdochium* and *Septoria*, as known in other *Bacillus* strains [4,5,42,43]. These observations are promising of a possible used for biocontrol of pathogenic fungi of cereals, particularly those producing mycotoxins such as *Fusarium* species. However, even if these capacities seem promising under laboratory conditions, it is now well known that the expression of microbial strains properties is mainly driven by environmental conditions after their application on fields. The confirmation of its biocontrol activity must be confirmed with *in planta* tests, under greenhouse or field trials conditions [44]. Rarely investigated in *Bacillus velezensis* strains considered for agriculture, antibiotics resistance patterns have been described for some food strains [45,46] and are very similar in gene contents and expression to the results shown in the present study. The genetic comparisons carried out here also found similar genes for a potential antibiotic resistance with close strains [33,34,35]. This is an important aspect for the regulatory process to describe which antibiotics resistance could be present and at which level they are expressed, as well as antibiotics sensitivity. In the present study, *Bacillus velezensis* strain B25 was shown to be able to produce auxin indole acetic acid, siderophores and to solubilize phosphorous from phosphate, which are common characteristics of this species [4,5]. We also showed that B25 is able to metabolize and grow on most single carbon sources as well as up to 8% NaCl and at pH5 and pH6. This information could help to refine optimal growth conditions in industrial production.

Another factor, almost never retrieved or linked with studies performed on the evaluation of the potential of microbial strains for biocontrol activity, is their ecotoxicological assessment [47]. Indeed, even the best microbial candidates to fight against a disease will be put aside, if their environmental impact is not compatible with the regulation. As the biocontrol active substances and products are currently evaluated at the European level on the same basis than the chemical compounds, the list of toxicological, ecotoxicological and residues in environment studies to provide is important and represent thereby an important point to consider, even before large campaigns of efficacy trials. Some of these studies remain affordable and represent an advantageous approach of the product impacts on major environmental compartments, such as the one performed in this study on *Eisenia fetida* and *Daphnia magna* [48,49]. In our hands, the commercial formulation of the B25 strain showed no effects on these two organisms. The crossed-knowledge between the absence of ecotoxicological impact of strain B25 and the fact that close neighbors’ strains have already been evaluated and added on the positive list of active substances for plant protection (i.e., *Bacillus amyloliquefaciens* FZB24, *Bacillus amyloliquefaciens* MBI600, *Bacillus amyloliquefaciens spp. plantarum* D747, *Bacillus subtilis* QST 713, *Bacillus subtilis* IAB BS03) trace the path for *Bacillus velezensis* strain B25 registration as a plant protection agent.

## 5. Conclusions

In summary, we described the genetic characteristics of *Bacillus velezensis* strain B25, and provided genetic comparison with close strains. Its properties as a plant biostimulant and biocontrol agent have been investigated through biochemical assays and in vivo assays. *Bacillus velezensis* strain B25 proved to be less UV sensitive than the neighbor strain IT45, which could be an asset in aerial uses in agriculture. It owns genes for potential production of secondary antibacterial metabolites and antifungal enzymes. It expressed a strong antifungal effect in vitro against a range of fungal pathogens of cereals. An in vivo co-inoculation of strain B25 and the fungal pathogen *Fusarium graminearum* showed a 30% decrease of the disease severity. Moreover, the studies performed on *Eisenia fetida* and *Daphnia magna* revealed the absence of effects of strain B25 on these organisms. These findings could therefore open the way for entering in the registration process as a plant protection agent.

## Figures and Tables

**Table 1:** Genomic features of *Bacillus velezensis* strain B25 and comparison with others *Bacillus* spp.

	*Bacillus velezensis* B25	*Bacillus methylotrophicus* FZB42	*Bacillus amyloliquefaciens* IT-45	*Bacillus subtilis* 168	*Bacillus licheniformis* ATCC14580
Genome size (bp)	3,854,619	3,918,589	3,928,857	4,214,630	4,222,336
G+C content (%)	46.71	46.4	46.62	43.5	46.2
Protein-coding sequences	3683	3693	3949	4106	4208
Average CDS size (bp)	934	895	888	896	873
Percentage of coding region	89	88	89	87	86
Ribosomal RNA operons	7	10	10	10	7
Number of tRNAs	68	89	95	86	72
Reference study and GenBank accession number	Gerbore et al. [16] LN999829.1	Chen et al. [33] CP000560.2	Tompkins et al. [34] CP004065.1	Kunst et al. [35] AL009126.3	Rey et al. [36] CP000002.3

**Table 2:** Gene clusters involved in synthesis of secondary metabolites in *B. velezensis* strain B25. NRPS: nonribosomal peptide, PKS: polyketide synthase, RP: ribosomal peptide.

Compound	Enzyme	Gene	High Similarity with	Reference Study
Surfactin	NRPS	srfABCD	FZB42	Chen et al. [33]
Fengycin	NRPS	fenABCDE	FZB42	
Bacillibactin	NRPS	dhbABCDEF	FZB42	
Bacilysin	NRPS	bacABCDEFG	FZB42	
Macrolactin	PKS	minABCDEFGHI	FZB42	
Bacillaene	PKS/NRPS	baeBCDE, acpK, baeGHIJLMNRS	FZB42	
Difficidin	PKS	dfnAYXBCDEFGHIJKLM	FZB42	
Mycosubtilin	NRPS	mycABC	IT-45	Tompkins et al. [34]
Amylolysin	RP	amyIEKRAMT	GA1	Arguelles-Arias [37]

**Table 3:** Antibiotic resistance genes detected in *Bacillus velezensis* strain B25 genome.

B25 Gene Label	Gene Symbol	Product	Antibiotic Class	Similarity with (Amino Acid Identity %)
BAMMD1_0254	lmrB	Lincomycin resistance protein	lincosamide	IT45 (99.8%), FZB42 (99.4%), B168 (88.4%)
BAMMD1_0286	Mdr	multidrug-efflux transporter	aminonucleoside, fluoroquinolone	IT45 (100%), FZB42 (99.6%), B168 (90.2%)
BAMMD1_0294	tmrB	Tunicamycin resistance protein	nucleoside	IT45 (98.5%), FZB42 (98.5%), B168 (77%)
BAMMD1_0514	Cfr	Ribosomal RNA large subunit methyltransferase	oxazolidinone, lincosamide, streptogramin, phenicol	IT45 (99.7%), FZB42 (97.4%)
BAMMD1_0534	vmlR	Ribosome protection protein	streptogramin, oxazolidinone, lincosamide, pleuromutilin, phenicol, tetracycline, macrolide	IT45 (100%), FZB42 (98.3%), B168 (71%)
BAMMD1_0665	aadK	Aminoglycoside 6-adenylyltransferase	aminoglycoside	IT45 (99.3%), B168 (63%)
BAMMD1_1112	Bla	Metallo-beta-lactamase type 2	Beta-lactam	IT45 (99.6%), FZB42 (97.2%)
BAMMD1_1128	penN	Beta-lactamase	Beta-lactam	IT45 (99.7%), FZB42 (95.7%), B168 (88.5%)
BAMMD1_2341	tetB	Tetracycline resistance protein	tetracycline	IT45 (99.3%), B168 (86.7%)

**Table 4:** Evaluation *B. velezensis* strain B25 biocontrol effect on fusarium seedling blight (FSB) syndrome caused by *Fusarium graminearum* B377. Different letters above the values (means ± standard deviation, *n* = 30) show significant differences in FSB disease severity according to one-way ANOVA, followed by Tukey post-hoc test.

Treatment	FSB Disease Severity	
	Test 1	Test 2
Control	0.9 (± 0.31) ^ab^	0 (± 0) ^a^
*Bacillus velezensis* B25	0 (± 0) ^a^	0.12 (± 0.12) ^a^
*Fusarium graminearum* B377	6.5 (± 1.33) ^c^	8.38 (± 1.51) ^b^
B25 + B377	4.41(± 1.1) ^bc^	4.59 (± 0.96) ^c^

**Table 5:** Metabolic profile of B25 on Biolog GEN III microplate: (+) growth, (−) no growth.

Substrates of Biolog Gen III					
Negative Control	−	D-Lactic Acid Methyl Ester	+	l-Aspartic Acid	+
Dextrin	+	l-Lactic Acid	+	l-Glutamic Acid	+
d-Maltose	+	Citric Acid	+	l-Histidine	+
d-trehalose	+	α-Keto-Glutaric Acid	+	l-Pyroglutamic Acid	+
d-Cellobiose	+	d-Malic Acid	+	l-Serine	+
Gentiobiose	+	l-Malic Acid	+	Pectin	+
Sucrose	+	Bromo-Succinic Acid	+	d-Galacturonic Acid	+
d-Turanose	+	Tween 40	+	l-Galactonic Acid Lactone	+
Stachyose	+	ϒ-Amino-Butyric Acid	+	Positive control	+
d-Raffinose	+	α-Hydroxy-Butyric Acid	−	pH 6	+
α-d-Lactose	+	β-Hydroxy-d, l-Butyric Acid	+	pH 5	+
d-Melibiose	+	α-Keto-Butyric Acid	−	1% NaCl	+
β-Methyl-d-Glucoside	+	Acetoacetic Acid	+	4% NaCl	+
d-Salicin	+	Propionic Acid	+	8% NaCl	+
*N*-Acetyl-d-Glucosamine	+	Acetic Acid	+	1% Sodium Lactate	+
*N*-Acetyl-d-Mannosamine	+	Formic Acid	+	Fusidic acid	−
*N*-Acetyl-d-Galactosamine	+	l-Fucose	+	d-Serine-2	−
*N*-Acetyl Neuraminic Acid	+	l-Rhamnose	+	Troleandomycin	−
α-d-Glucose	+	Inosine	+	Rifamycin SV	−
d-Mannose	+	d-Sorbitol	+	Minocycline	−
d-Fructose	+	d-Mannitol	+	Lincomycin	+
d-Galactose	+	d-Arabitol	+	Guanidine HCl	+
3-Methyl Glucose	+	Myo-inositol	+	Niaproof 4	−
d-Fucose	+	Glycerol	+	Vancomycin	−
d-Gluconic Acid	+	d-Glucose-6-PO4	+	Tetrazolium violet	+
d-Glucuronic Acid	+	d-Fructose-6-PO4	+	Tetrazolium Blue	−
Glucuronamide	+	d-Aspartic Acid	+	Nalidixic Acid	−
Music Acid	+	d-Serine	−	Lithium Chloride	+
Quinic Acid	+	Gelatin	+	Potassium Tellurite	+
d-Saccharic Acid	+	Glycyl-l-Proline	+	Aztreonam	−
*p*-Hydroxy-Phenylacetic Acid	−	l-Alanine	+	Sodium Butyrate	+
Methyl pyruvate	+	l-Arginine	+	Sodium Bromate	−

**Table 6:** Minimal inhibitory concentrations (MICs) of antibiotics obtained for *Bacillus methylotrophicus* strain B25.

Antibiotics	Imipenem	Amikacin	Ceftazidime	Ciprofloxacin	Vancomycin	Erythromycin	Tetracycline
standard range [µg·mL^−1^]	[32–0.002]	[256–0.015]	[256–0.015]	[32–0.002]	[256–0.015]	[256–0.015]	[256–0.015]
*MIC for* *Bacillus B25* [µg·mL^−1^]	0.015	1	32	0.03	0.5	0.12	4

**Table 7:** Sensitivity of *Bacillus* strains to UV-C.

	Exposure Time under UV-C (Minutes)						
Bacterial strains	0	5	10	15	20	25	30
*Bacillus* B25	+	+	+	−	−	−	−
*Bacillus* IT45	+	−	−	−	−	−	−

**Table 8:** *Bacillus velezensis* strain B25 effects on mortality, biomass increase, and fecundity of *Eisenia fetida.* NOEC stands for no observed effect concentration and EC50 for half maximal effective concentration.

Product Tested	*Bacillus velezensis* Strain B25 (1 × 10^8^ cfu·g^−1^)			
Assay Element/Substrate	*Eisenia fetida*/Artificial Soil			
Application Rates (kg·ha^−1^)	Average Mortality at 28 Days (%) *	Biomass Increase (Average %) **	Fecundity	
			Average Number per Adult	Effects (%) ***
Control	0	+80.69	17.84	NA
1	1.30 (NS)	+87.20 (NS)	16.93 (NS)	−5.63 (NS)
2	−3.90 (NS)	+86.84 (NS)	14.33 (NS)	−20.14 (NS)
10	−3.90 (NS)	+85.51 (NS)	17.15 (NS)	−4.39 (NS)
**NOEC (kg·ha^−1^)**	10			
**EC_50_ (kg·ha^−1^)**	>10			

* Based on the average of dead or not retrieved organisms, positive values indicate a higher mortality compared to control group, negative values indicate a lower mortality compared to control group. ** Positive values indicate an increase of biomass in comparison with day 0. *** Positive values indicate a higher fecundity compared to control group; negative values indicate a lower fecundity compared to control group. Significant differences (S) or non-significant differences (NS) based on Kruskal–Wallis test for mortality values and ANOVA for biomass increase and fecundity. NA = Not applicable.

**Table 9:** *Bacillus velezensis* Strain B25 Effects on immobilization, body length, and reproduction of *Daphnia Magna*.

Endpoint		Concentration (mg·L^−1^)
Immobilization	EC_10_ (95% confidence limits)	2853 (Not determined)
Immobilization	EC_20_ (95% confidence limits)	4484 (Not determined)
Immobilization	EC_50_ (95% confidence limits)	9712 (Not determined)
No Observed Effect Concentration		4500
Lowest Observed Effect Concentration		10,000
Body length	EC_10_ (95% confidence limits)	5963 (4413 to 8058)
Body length	EC_20_ (95% confidence limits)	>10,000 (Not determined)
Body length	EC_50_ (95% confidence limits)	>10,000 (Not determined)
No Observed Effect Concentration		2200
Lowest Observed Effect Concentration		4500
Reproduction	EC_10_ (95% confidence limits)	4240 (Not determined)
Reproduction	EC_20_ (95% confidence limits)	5062 (Not determined)
Reproduction	EC_50_ (95% confidence limits)	6855 (Not determined)
No Observed Effect Concentration		4500
Lowest Observed Effect Concentration		10,000

## Data Availability

The data presented in this study are available in supplementary material and on request from the corresponding author.

## Supplementary Materials

The following are available online at https://www.mdpi.com/article/10.3390/microorganisms9091924/s1, Table S1: *Bacillus velezensis* strain B25 genes coding for xylanase and glucanase enzymes; Figure S1: Back view (left) and front view (right) of challenge co-cultures of *Bacillus velezensis* B25 against pathogenic fungi (i) *Gaeummannomyces graminis* B278; (ii) *Fusarium graminearum* B375; (iii) *Fusarium culmorum* B376; (iv) *Fusarium graminearum* B377, (v) *Fusarium moniliforme* B378; (vi) *Microdochium nivale* B379; (vii) *Septoria nodorum* B380; (viii) *Fusarium graminearum* FG183 B381; (ix) *Fusarium graminearum* FG171 B382; (x) *Fusarium graminearum* B383; (xi) *Fusarium verticillioides* FV63 B384; (xii) *Fusarium graminearum* FG 155 B385 and (xiii) *Fusarium verticillioides* FV838 B386; Figure S2: Screening for siderophores production ability of *Bacillus velezensis* B25: Control-1 non-inoculated (left), test (middle), Control-2 B25 without O-CAS (right).
