# Supplementary material for: From Strain Characterization to Field Authorization: Highlights on Bacillus velezensis Strain B25 Beneficial Properties for Plants and Its Activities on Phytopathogenic Fungi
Source: Microorganisms. 2021 Sep 10;9(9):1924. doi: 10.3390/microorganisms9091924 (PMC8472612; doi:10.3390/microorganisms9091924)
Supplement: Supplementary file 1 [file microorganisms-09-01924-s001.zip › microorganisms-1316929-supplementary.pdf]

Table S1. *Bacillus velezensis* strain B25 genes coding for xylanase and glucanase enzymes.

Label	Gene	Length	Product	EC number
BAMMD1_1760	xynC	1272	endo-xylanase	3.2.1.136
BAMMD1_3310	xynA	642	endo-1,4-beta-xylanase	3.2.1.8
BAMMD1_1755	eglS	1500	endo-1,4-beta-glucanase	3.2.1.4
BAMMD1_3541	bglS	732	endo-beta-1,3-1,4 glucanase	3.2.1.73, 3.2.1.6

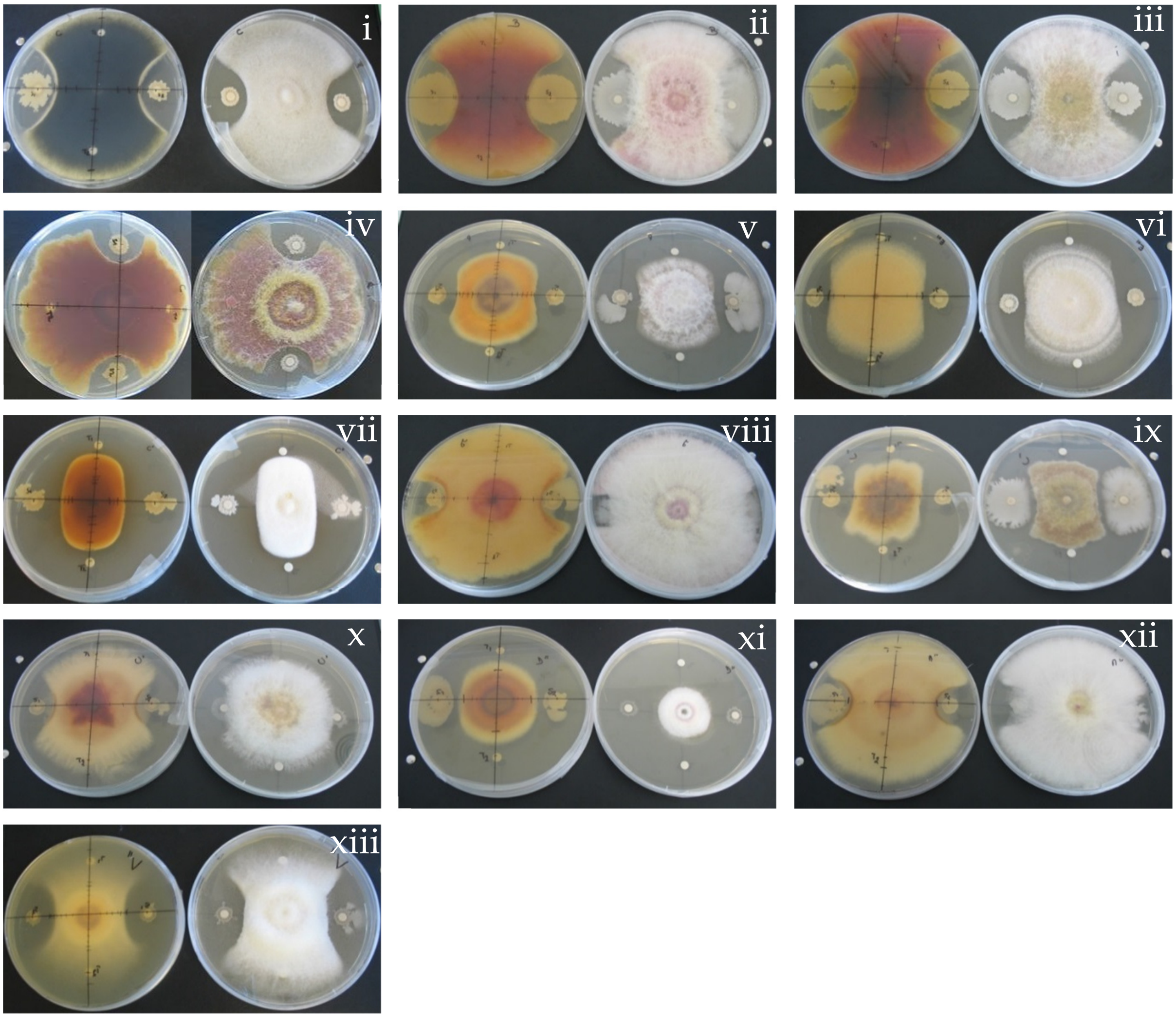

Figure S1. Back view (left) and front view (right) of challenge co-cultures of *Bacillus velezensis* B25 against pathogenic fungi (i) *Gaeummannomyces graminis* B278; (ii) *Fusarium graminearum* B375; (iii) *Fusarium culmorum* B376; (iv) *Fusarium graminearum* B377, (v) *Fusarium moniliforme* B378; (vi) *Microdochium nivale* B379; (vii) *Septoria nodorum* B380; (viii) *Fusarium graminearum* FG183 B381; (ix) *Fusarium graminearum* FG171 B382; (x) *Fusarium graminearum* B383; (xi) *Fusarium verticillioides* FV63 B384; (xii) *Fusarium graminearum* FG 155 B385 and (xiii) *Fusarium verticillioides* FV838 B386.

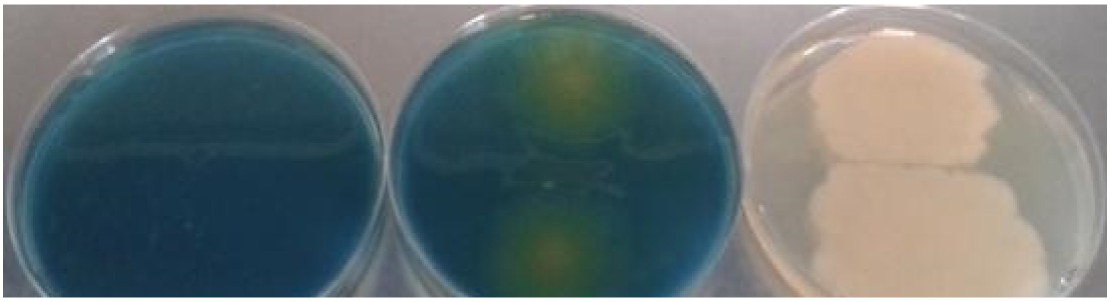

Figure S2. Screening for siderophores production ability of *Bacillus velezensis* B25: Control-1 non-inoculated (left), test (middle), Control-2 B25 without O-CAS (right).
